# Clinical Relevance of Informal Coercion in Psychiatric Treatment—A Systematic Review

**DOI:** 10.3389/fpsyt.2016.00197

**Published:** 2016-12-12

**Authors:** Florian Hotzy, Matthias Jaeger

**Affiliations:** ^1^Department for Psychiatry, Psychotherapy and Psychosomatics, University Hospital of Psychiatry Zurich, Zurich, Switzerland

**Keywords:** informal coercion, leverage, attitudes, prevalence, clinical effect, mental health, therapeutic relationship

## Abstract

**Introduction:**

Although informal coercion is frequently applied in psychiatry, its use is discussed controversially. This systematic review aimed to summarize literature on attitudes toward informal coercion, its prevalence, and clinical effects.

**Methods:**

A systematic search of PubMed, Embase, PsycINF, and Google Scholar was conducted. Publications were included if they reported original data describing patients’ and clinicians’ attitudes toward and prevalence rates or clinical effects of informal coercion.

**Results:**

Twenty-one publications out of a total of 162 articles met the inclusion criteria. Most publications focused on leverage and inducements rather than persuasion and threat. Prevalence rates of informal coercion were 29–59%, comparable on different study sites and in different settings. The majority of mental health professionals as well as one-third to two-third of the psychiatric patients had positive attitudes, even if there was personal experience of informal coercion. We found no study evaluating the clinical effect of informal coercion in an experimental study design.

**Discussion:**

Cultural and ethical aspects are associated with the attitudes and prevalence rates. The clinical effect of informal coercion remains unclear and further studies are needed to evaluate these interventions and the effect on therapeutic relationship and clinical outcome. It can be hypothesized that informal coercion may lead to better adherence and clinical outcome but also to strains in the therapeutic relationship. It is recommendable to establish structured education about informal coercion and sensitize mental health professionals for its potential for adverse effects in clinical routine practice.

## Introduction

Informal coercion is ubiquitous in the health-care system, especially in mental health and psychosocial services. It comprises a large range of treatment pressures and interventions that can be applied by the professional with the intention to foster treatment adherence or avoid formal coercion. The degree of coercion adherent to several interventions ranges between full autonomy and formal coercion that is regulated by the law. Generally, informal coercion is intertwined with the therapeutic relationship and frequently applied by the professional unintentionally (1). The intensity of coercion that is perceived by the patient consecutively interacts with various aspects, such as transparency, fairness, dignity, trust, and the quality of the therapeutic alliance itself (2). Therefore, perceived coercion does not necessarily correlate with factual coercion, both formal and informal (3, 4).

The spectrum of informal coercive measures constitutes a continuum of phenomena, ranging from subtle interpersonal interactions to obvious demonstrations of force. Several graduations of informal coercion have been described, and the most commonly used categorizations are as follows: Szmukler and Appelbaum (5) defined a hierarchy of treatment pressures with: (I) persuasion; (II) interpersonal leverage; (III) inducements; (IV) threats; and (V) compulsory treatment. More detailed, Lidz et al. (6) defined nine graduations of coercion: (I) persuasion, (II) inducement, (III) threats, (IV) show of force, (V) physical force, (VI) legal force, (VII) request for a dispositional preference, (VIII) giving orders, and (IX) deception.

Beyond full autonomy, persuasion and conviction are the least problematic interventions on the spectrum of treatment pressures as it relies on respect for the patient’s values and arguments. It is a very common phenomenon in the interaction of patients and professionals and is also compatible with a therapeutic relationship that aims at an informed consent and a shared decision-making process (7). Persuasion can be differentiated from conviction by the nuance that conviction targets on the result that the patient comes to own conclusions during a reciprocal discussion while persuasion results in the adoption of the professional’s opinion by the patient.

Ascending in the hierarchy of treatment pressures, the notion of professional force becomes more obvious resulting in a more asymmetrical therapeutic relationship. There is a range of utilitarian interventions that are applicable on the basis of emotional or factual dependency of the patient within the professional relation. Interpersonal leverage may occur if the patient shows emotional dependency on the professional which may be used for interpersonal pressure. The clinician expresses verbally or non-verbally his or her expectations or demonstrates disappointment. The patient is tempted to react in a way that he or she assumes would please the clinician. A more factual form of leverage is the use of inducements within a framework of negotiation. Thereby, the patient is demanded to comply with treatment in exchange for a desired asset. Several goods or values can be used as leverage tools. Monahan et al. (8) described four specific types of leverage: housing, money, children, and criminal justice. Other types, such as work, non-monetary goods, attention, and care, are probably common in the health-care system as well. A fluent transition from offer to threat in this context seems obvious. A distinction can be made considering the normative basic entitlement. If the patient could receive a desired good in addition to standard care or basic rights, it can be called an offer. If some basic right or standard good is withheld from the patient, it is considered a threat (9). Thus, the classification of the proposition made by the professional strongly depends on the factual, legal, or moral baseline (10, 11). Although the differentiation between offer and threat may be difficult within the spectrum of leverage tools, there are a range of obvious threats comprising a more subtle demonstration of force up to announcement of negative sanctions.

To date, there is no comprehensive digest on informal coercion in mental health-care systems. Therefore, the aim of this study was to review the literature on prevalence of treatment pressures and informal coercion and the attitudes toward and clinical consequences of these interventions from the perspective of patients and professionals.

Our hypothesis was that evidence on informal coercion is scarce and mainly refers to prevalence and attitudes rather than to clinical effects. Additionally, we hypothesized that the literature relies mostly on cross-sectional studies, and studies of higher quality such as randomized controlled trials would be absent due to the complexity of operationalization and ethical reasons that might prevent an interventional study.

## Materials and Methods

**Figure 1:**
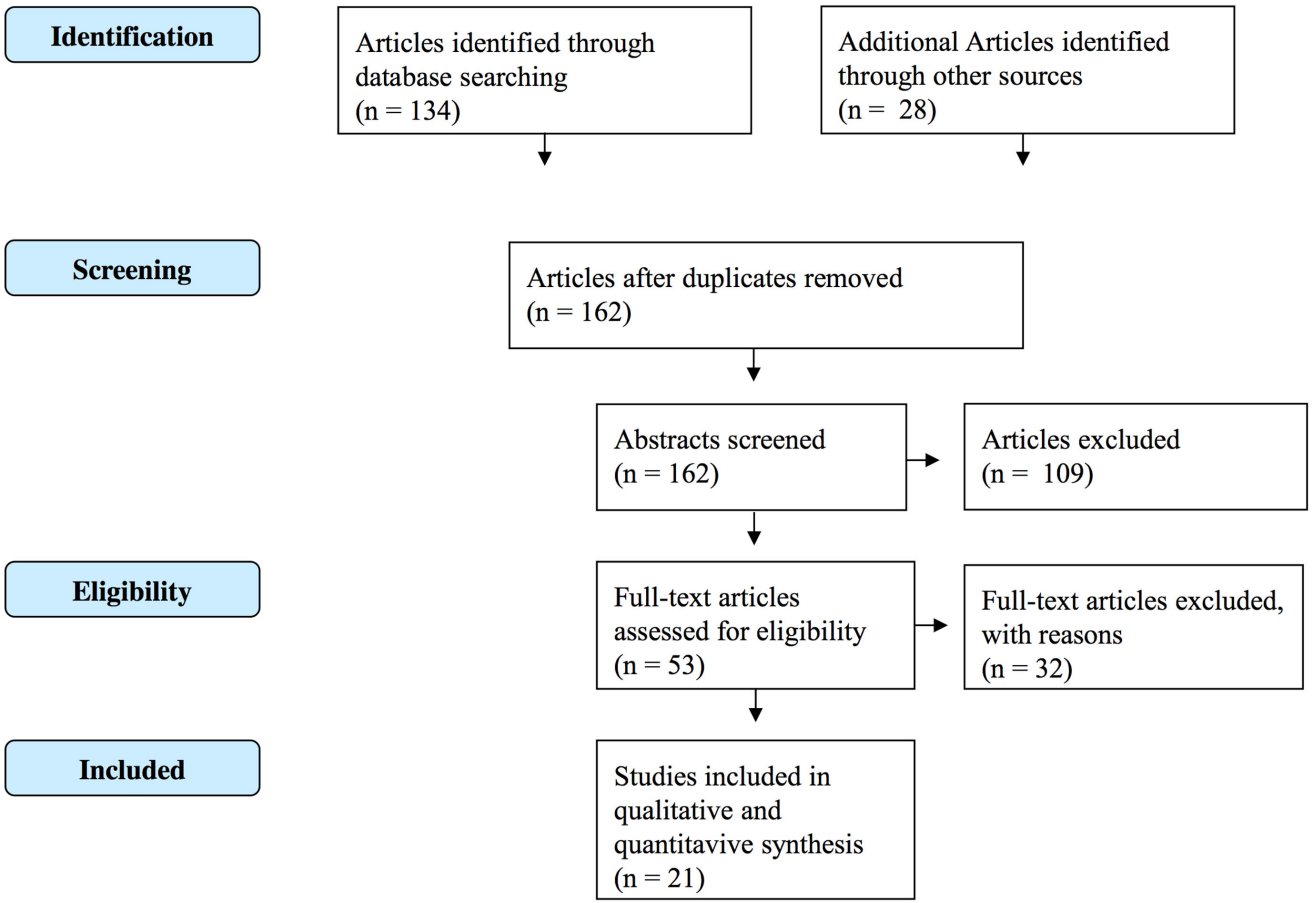
**Prisma-based flow diagram**.

A systematic strategy was used to search the electronic databases PubMed/Medline, Embase, PsycINFO, and Google Scholar for studies published after the year 2000. A subject and text word search strategy was used with the words “informal coercion,” OR “treatment pressure,” OR leverage. Those words were combined with psychiatr* OR psychiatry OR “mental health.” References of the included studies and other reviews related to this topic were also inspected and relevant articles were included (referred to as “other sources” in Figure 1).

### Inclusion and Exclusion Criteria

Studies containing original data describing patients’ and/or clinicians’ attitudes toward informal coercion were included. Also studies evaluating the prevalence and clinical effects of informal coercion were included. Regarding the low number of publications in this very specific topic, no quality threshold for inclusion was set. Only studies published after the year 2000 were included. Relevant articles were filtered according to the Prisma-statement (12) (Figure 1). Studies focusing on formal/legal coercion were excluded except when they did have explicit aims to investigate informal coercion in the context of legally involuntary in- or outpatient treatment. Studies on perceived coercion were included when conducted in the context of informal coercion, but excluded in the context of formal coercion.

### Analysis

The following categories were built to classify studies by themes. (I) Attitudes of staff to informal coercion in in- and outpatient settings. (II) Attitudes of patients toward informal coercion in in- and outpatient settings. (III) Prevalence of informal coercion. (IV) Clinical effects/aspects of informal coercion.

The quality of the included studies was assessed according to a hierarchy of evidence (categorizing studies by the attributes of their design) and the relevance for the topic as described in the results chapter. The results are partially categorized and summarized in a narrative way.

## Results

The search procedure yielded 162 articles. Of these, 21 met the inclusion criteria (Figure 1). The 21 publications referred to 15 studies [1 study resulted in 2 (13, 14), another study in 6 publications (15–20), and 13 studies in 1 publication (21–33)].

### Quality of the Studies Included

All studies included were cross sectional (Table 1). No experimental or quasi-experimental studies were found. Four studies assessed mental health professionals only, thereof two using focus group interviews, one using case vignettes and one using structured interviews. Two studies used focus groups with professionals and patients. Nine studies assessed patients only, most of them using structured individual interviews, and one using focus groups, another using individual qualitative interviews. The sample sizes varied between 24 and 1,011 participants. Professionals were from several settings including in- and outpatient services, ACT, and housing institutions. Most patients were recruited in outpatient setting, three studies also included inpatients.

**Table 1:** **Study characteristics (*N* = 15)**.

Reference	Design	Participants	Sample size	Clinical setting	Outcome measure	Country/state/city
**Study population: professionals**						

Valenti et al. (21)	Qualitative design using focus groups	Mental health professionals	248	Inpatient and outpatient	Attitudes and experiences	10 countries^a^

Jaeger et al. (22)	Quantitative design using questionnaires with case vignettes	Mental health professionals	39	Inpatient	Attitudes and experience; attribution of degree of coercion	Switzerland

Rugkasa et al. (23)	Qualitative design using focus groups	Mental health professionals	48	Community mental health services	Attitudes and experiences	UK

Wong et al. (24)	Quantitative design using structured interviews	Staff in housing institutions	27	Housing institutions	Attitudes and prevalence of housing as leverage	Pennsylvania

**Study population: professionals and patients**						

Priebe et al. (25)	Qualitative design using focus groups	Mental health professionals, other mental health service stakeholders, and patients	Professionals: 92 Patients: 27 Other: 20	Outpatients	Attitudes on money as leverage tool	UK

Appelbaum and Le Melle (26)	Qualitative design using focus groups	Mental health professionals and patients	Professionals: 23 Patients: 21	ACT services	Attitudes and experiences	New York

**Study population: patients**						

Norvoll and Pedersen (27)	Qualitative design using focus groups	Patients	24	Inpatient and outpatient	Attitudes	Norway

Canvin et al. (28)	Qualitative design using semi-structured interviews	Patients	29	Outpatient	Attitudes	UK

Burns et al. (29)	Quantitative design using structured interviews	Patients	417	Outpatient	Prevalence and patterns of leverage; Comparison to a US sample	UK

Jaeger and Rossler (13, 14)	Quantitative design using structured interviews	Patients	187	Inpatient and outpatient	Prevalence of several leverage tools, attitudes, perceived coercion	Switzerland

McNiel et al. (30)^b^	Quantitative design using structured interviews	Patients	198	Outpatient	Influence of leverage on treatment relationship and adherence	San Francisco

Angell et al. (31)^b^	Quantitative design using structured interviews	Patients	201	Outpatient	Influence of money as leverage tool on treatment relationship	Chicago

Redlich et al. (15) Robbins et al. (16) Swanson et al. (17) Appelbaum and Redlich (18) Van Dorn et al. (19) Monahan et al. (20)	Quantitative design using structured interviews	Patients	1,011	Outpatient	Prevalence of several leverage tools In consequent publications: prevalence of money as leverage tool; prevalence of housing as leverage tool; prevalence of leverage in patients with violent behavior	5 states in the US^c^

Elbogen et al. (32)	Quantitative design using structured interviews	Patients	104	Outpatient	Attitude on money as leverage tool	North Carolina

Elbogen et al. (33)	Quantitative design using structured interviews	Involuntary admitted patients	258	Inpatient	Perceptions of financial coercion	US

*^a^Canada, UK, Croatia, Germany, Chile, Mexico, Italy, Spain, Norway, and Sweden*.

*^b^Publication refers to a single subgroup of the US multicentre study (20)*.

*^c^Chicago, IL, USA; Durham, NC, USA; San Francisco, CA, USA; Tampa, FL, USA; Worcester, MA, USA*.

All publications had explicit *a priori* aims, and 17 discussed their data in the context of generalizability. None of the studies used a sample size calculation or justified the number of participants (Table 2). None of the studies declared dropouts. The nature of funding sources was disclosed in 13 out of 21 publications. The questionnaires were described conclusively in all publications that used structured interviews. Most studies assessed demographic parameters of the participants. Some studies found and discussed cultural differences in the prevalence of informal coercion and discussed these findings. One study especially aimed to investigate cultural differences in the prevalence of informal coercion between the UK and the US.

**Table 2:** **Qualitative evaluation of the included publications (*N* = 21)**.

Study	Explicit *a priori* aim	Sample size calculation	Inclusion/exclusion criteria stated	Research independent of routine care/practice	Original questionnaire available	Response/dropout rate specified	Discussion of generalizability	Demographic data	Cultural differences	Funding disclosed
Valenti et al. (21)	+	−	−	+	−	−	−	−	+	−

Jaeger et al. (22)	+	−	+	+	+	+	+	+	−	−

Rugkasa et al. (23)	+	−	+	+	−	−	+	−	−	+

Wong et al. (24)	+	−	−	+	−	−	+	+	−	+

Priebe et al. (25)	+	−	−	+	−	−	+	+	−	+

Appelbaum and Le Melle (26)	+	−	+	+	−	+	+	+	−	+

Norvoll and Pedersen (27)	+	−	+	+	−	−	+	+	−	−

Canvin et al. (28)	+	−	+	+	−	−	+	+	−	+

Burns et al. (29)	+	−	+	−	+	+	+	+	+	−

Jaeger and Rossler (13)	+	−	+	−	+	−	+	+	−	−

Jaeger and Rossler (14)	+	−	+	−	+	−	+	+	+	−

McNiel et al. (30)	+	−	+	−	+	−	−	+	+	+

Angell et al. (31)	+	−	+	−	+	−	+	+	−	+

Redlich et al. (15)	+	−	+	−	+	+	+	+	+	+

Robbins et al. (16)	+	−	+	−	+	−	+	+	+	−

Swanson et al. (17)	+	−	+	−	+	−	+	+	+	+

Appelbaum and Redlich (18)	+	−	+	−	+	−	−	+	+	+

Van Dorn et al. (19)	+	−	+	−	+	+	+	+	+	+

Monahan et al. (20)	+	−	+	−	+	−	+	+	+	+

Elbogen et al. (32)	+	−	+	−	+	−	+	+	−	−

Elbogen et al. (33)	+	−	+	−	+	+	+	+	+	+

Almost all studies examined attitudes toward informal coercion. Four studies, one from the UK, two from the US, and one from Switzerland, evaluated the prevalence of several interventions comprising informal coercion, mostly leverage tools. Most of the studies examined leverage as one form of informal coercion in one of the following categories: housing, justice, childcare, employment, and money. Studies searching for informal coercion in general or leverage without categorization were rare. There were no interventional studies assessing the clinical effect of informal coercion.

### The Perspective of Mental Health Professionals on Informal Coercion

Studies assessing mental health professionals did not evaluate specific prevalence rates. The four publications consistently stated that “most” of the professionals used informal coercion in daily routine practice (Table 3). The study investigating housing facilities found that about 60% of malcompliant residents were excluded from the program suggesting a frequent and incisive use of pressure to treatment adherence (24). Professionals intended to foster their patients’ ability to take responsibility for their lives and considered informal coercion as a justifiable method to reach this goal (23). Concerning clinical effects, participants considered informal coercion to be effective in the therapeutic process with respect to promotion of adherence resulting in avoidance of decompensation as well as formal coercion. Nevertheless, one study revealed that interventions with stronger informal coercion were less accepted by mental health professionals (22), and mental health professionals tended to avoid informal coercion and to respect the patients’ decisions if possible although some stated to feel being pressured to use it. Some participants used informal coercion more often than they were aware to use it (21), and one study revealed that the degree of coercion was underestimated in the whole study population. Detailed analysis showed differences in the underestimation of professions with physicians showing the least underestimation of the degree of coercion followed by nurses and other professions (22). Telling patients what to do, being judgmental, and threatening them were rated as the least successful methods (26). If informal coercion was used in the framework of negotiation and asserting authority, it was referred to as suitable to reach treatment goals.

**Table 3:** **Findings**.

Study	Prevalence	Attitudes	Clinical effect
**Study population: professionals**			

Valenti et al. (21)	Most participants used informal coercion	Rather positive, effective tool, participants feel pressured to use informal coercion and describe unpleasant feelings when it is used	Promotion of adherence, avoid formal coercion

Jaeger et al. (22)	–	Higher degrees of informal coercion were grossly underestimated but less accepted; participants with a negative attitude toward informal coercion overestimated the degree of coercion A trend to differences between professional groups	–

Rugkasa et al. (23)	Most participants used informal coercion	Necessary tool to achieve treatment goals	Informal coercion may lead to promotion of adherence and achievement of a healthy live Potential threat to relationships

Wong et al. (24)	59% of the supported independent living residents who refused to take prescribed medication resulting in decompensation were excluded from the program	Most programs considered medication non-compliance to be unacceptable when it resulted in decompensation Consumption of alcohol and/or other drugs and inviting other people was not accepted by most programs	Informal coercion helps to avoid decompensation

**Study population: professionals and patients**			

Priebe et al. (25)	–	Use of financial incentives is likely to raise similar concerns (e.g., value of medication, source of funding, how patients would use the money, effectiveness, impact on therapeutic relationship) in most stakeholders	Unclear responsibilities for potentially harmful medication effects, especially in the long term

Appelbaum and Le Melle (26)	Little evidence of significant use of leverage or perceptions of coercion	Staff and patients had quite similar opinions about treatment methods with supporting patients and building relationships being preferred mechanisms Few patients identified the least effective methods as scare tactics, threats and violating patients’ personal space	Importance of constant reflection over staff behavior to recognize unintended use of informal coercion

**Study population: patients**			

Informal coercion in general			

Norvoll and Pedersen (27)	Coercion unfolds in health, child and social services, which, when acting together, contribute to increasing the coercive pressure of compliance	Gray zone between formal and informal coercion How extensive, negative or legitimate coercion is viewed depends on several aspects before, during, and after the coercive incidents Strong impact of coercive measures on the patients self and identity Few participants saw informal coercion as helpful for their mental health problems and life situations	

Canvin et al. (28)	Participants experienced pressure not only from health professionals but also from family and friends and even themselves	Relationship with the mental health team was experienced as interpersonal pressure to accept treatment Three features of leveraged pressures: conditionality, a lever and direct communication	–

Burns et al. (29)	35% any leverage 24% housing 15% justice system 8% childcare 2% financial	–	Unable to draw any conclusions as to the efficacy of leverage

Jaeger and Rossler (13)	29% any leverage 19% housing 11% justice system 7% childcare 3% financial	Experience with informal coercion combined with a schizophrenic disorder was associated with higher perceived coercion; informal coercion was associated with lower perceived fairness; experience of informal coercion did not lead to different appraisal of its effectiveness; higher levels of perceived fairness and effectiveness were associated with higher insight into illness	–

Jaeger and Rossler (14)	29% any leverage 19% housing (55% of those who ever lived in supported housing) 11% justice system (27% of those with criminal sentence) 7% childcare (29% of those with children under the age of 16) 3% financial (8% of those with representative payee)	34–70% approved informal coercion in general, independently of own experience; justice system was the most and childcare the less approved form of informal coercion	–

McNiel et al. (30)	37% any leverage 17% housing 22% justice system 2% financial 3% outpatient commitment	Experience of leverage was not associated with medication adherence Higher treatment satisfaction was associated with a better working alliance, lower psychological reactance, and less perceived coercion	Better adherence to medication was associated with higher perceived coercion but also with a more positive experience of medication effects Benefits in medication adherence due to informal coercion may come at the cost of decreased treatment satisfaction on the basis of side effects

Redlich et al. (15)	41–55% any form of leverage 15–21% housing 11–23% justice system 3–7% childcare 6–20% financial 2–10% employment Health service providers were the most frequent source of pressures (49%), followed by family members and friends (28%)	–	–

Van Dorn et al. (19)	–	55–69% perceived treatment leverage to be fair 48–60% perceived leverage to be effective Patients with psychosis and high barriers to care tend to view leverage as unfair Patients with less perceived coercion and better insight believe that they benefit from formal and informal sanctions Participants with experience with leverage were significantly more likely to endorse its effectiveness whereas higher perceived coercion was associated with lower perceived effectiveness	–

Monahan et al. (20)	44–59% any leverage 23–40% housing 15–30% justice system 7–19% financial	–	–

Housing leverage			

Robbins et al. (16)	22–40% housing leverage In 43% the landlord applied housing leverage, in 29% mental health professionals, more seldom family (11%) or friends (6%), unstated rule (18%) or “self” (9%)	Housing leverage led to higher scores of perceived coercion but had no influence on treatment satisfaction Patients who experienced housing leverage rated its use to help people stay well more often than those without experience	–

Judicial leverage			

Swanson et al. (17)	Violent offenders had experienced leverage twice as likely as other patients Experience of both legal and social welfare leverage was significantly associated with higher rates of serious violence	–	Concerns about safety and non-adherence to treatment may influence clinicians and judges to apply legal leverage

Financial leverage			

Angell et al. (31)	53% of the patients had a payee or money manager, which was in 79% a clinician payee 40% of patients with a clinician payee perceived financial leverage	Respondents with clinician payees (relative to those with family or friend payees or no payees) reported more conflict in the therapeutic relationship but had no difference in their bond scores in comparison with the other respondents	Payeeship may lead to strain in the therapeutic relationship when it is used for promoting adherence

Appelbaum and Redlich (18)	31–53% ever had a representative payee Between 13 and 29% of those who had experienced financial leverage	No significant relationship between money leverage and treatment satisfaction Patients who experienced money leverage rated its use as effective more often than those without experience Those with a family member as the representative payee were more satisfied and felt significantly less pressure	–

Elbogen et al. (32)	–	Patients rated money as leverage helpful if they also felt that other pressures were helpful for improving adherence 81% of the patients found legal pressures as helpful to keep them in treatment 65% reported that withholding money was not a useful method to improve treatment adherence	The use of money as leverage to improve adherence can lead to disturbance of the therapeutic relationship

Elbogen et al. (33)	30% perceived financial leverage 14% of clinicians and family members reported giving money warnings	–	Perceived financial coercion is increased in the presence of other forms of mandated treatment

*In order to improve legibility, publications are listed in accordance with their topic*.

In summary, professionals rated informal coercion to be effective and useful in some situations, especially if it concerned interventions with less obvious and strong coercion. But the use of informal coercion was regarded as a critical intervention, and some participants stressed the importance of continuous reflection on the usage of informal coercion within treatment teams (to “keep each other in check”) (26) as well as individually. Possible alternatives, including less influence and coercion, were consistently favored. Nevertheless, it seems to be a frequently applied interventional approach within therapeutic interactions in psychiatric health care.

### The Perspective of Patients on Informal Coercion

As opposed to the studies focusing on professionals, some publications investigating patients’ perspective on informal coercion were able to number the prevalence among the samples. Similar to the studies focusing on the professional perspective, these publications mainly reported results concerning leverage, rather than other forms of informal coercion. Most studies investigated the prevalence of leverage tools in general as well as the prevalence of specific forms of leverage (Table 3). Money, housing, and work are used as leverage tools to induce treatment adherence within the social welfare system. An individual with mental disorder would only gain access to the desired support if psychiatric treatment, and/or medication, was accepted. In the context of the judicial system, similar circumstances might emerge when a psychiatric patient agrees to adhere to treatment in order to avoid prosecution or an unfavorable judicial order, such as incarceration. Individuals with children also might face restriction of their parental rights if they do not consent to psychiatric treatment. Twenty-nine to fifty-nine percent of the patients from several study sites reported the experience of any form of leverage. The lowest rates were found in Switzerland and the highest in the US. The most frequently used leverage tool was housing with rates from 15 to 40% of all patients. Financial leverage was reported by 2–30%. Employment was only assessed in one US study, and 2–10% of the study participants reported experience. The prevalence rates of judicial leverage tools ranged from 11 to 23% and childcare was used in 3–8%. Health-care providers were identified as the most prevalent sources of treatment pressures next to family members, friends, and payees among others. Canvin et al. (28) found that patients experienced pressure not solely in mental health care but in everyday life with family and friends.

Attitudes toward informal coercion were examined by most of the studies including general appraisal, evaluation of fairness, and effectiveness. Thirty-four to eighty-one percent of the patients described different forms of leverage as helpful and approved its usage independently of their own experience (14, 19). The particular forms of leverage were rated differently with justice as most approved and children as less approved form (14). In one US study, 55–69% of the patients perceived the use of leverage as fair and 48–60% as effective (19). In some publications, those patients who experienced informal coercion tended to rate its effectivity higher than those without experience of informal coercion (13, 14, 16, 18, 19). Controversially, some qualitative studies reported that only a few patients found coercion to be helpful (27), and informal coercion was rated as the less successful compared to interventions on a merely voluntary basis (26).

Some studies which tended to characterize the participants showed that informal coercion was rated more positive by patients with higher insight (13, 19) and less perceived coercion (19) whereas experience of informal coercion and a schizophrenic disorder were associated with higher perceived coercion scores and lower perceived fairness (13, 19).

No study aimed primarily to evaluate the clinical effect of informal coercion in an experimental or quasi-experimental setting. Only subjective ratings on the effectiveness of informal coercion were assessed in some studies as mentioned above.

## Discussion

### Prevalence of Informal Coercion

This systematic review shows that informal coercion is used as a method to enhance treatment adherence in different countries and with a high prevalence according to investigations among patients as well as professionals. Most frequently, different forms of leverage were evaluated rather than other interventions comprising informal coercion (i.e., persuasion, threat). One-third to half of the patients reported having been subjected to some sort of leverage within interactions in psychiatric therapy and care. Also, most of the professionals stated to use leverage and other forms of informal coercion within their therapeutic activities. The supported housing sector appeared to be associated the most with the use of leverage next to the criminal and civil justice sectors. Money and work were not as frequently reported as leverage tools. The most prevalent requirement to adhere to psychiatric treatment and medication for getting access to a supported housing facility might be regarded as structural informal coercion within the mental health-care system (34). The use of leverage within the justice system on the other hand works as a coercive informal admission to the mental health-care sector. Both pathways supposedly lead to an increased rate of patients who are at least not completely voluntarily in treatment. This routine link between mental health care and other societal sectors most likely contributes to the stigma that coercion is inherently attached to mental health care. Vice versa, this stigma of coercion might induce the use of the mental health-care system as a leverage tool to achieve non-medical aims. Nevertheless, informal coercion seems to result in a higher rate of psychiatric treatment of those in need (35) and to better outcome according to the opinions of both, patients and professionals.

### Attitudes toward Informal Coercion

Next to a rather high appraisal by patients and professionals of at least weaker forms of informal coercion, such as persuasion and leverage, the use of informal coercion was considered critical to interfere with the therapeutic relationship. If inducing high levels of perceived coercion and having a notion of unfairness, informal coercion might impede the therapeutic relationship and lead to dropouts from treatment (1). Moreover, by increasing the association of psychiatric care with the notion of coercion, informal coercion might result in avoidance of the mental health-care system of others (36). It is not known if the number of individuals conducted into the mental health-care system by informal coercion outweighs the number of those who refrain from mental health care due to fear of being subjected to coercion. Thus, the effect of informal coercion (as well as formal coercion) on public mental health and health-care costs is unknown.

If applied transparently and fairly, informal coercion was considered helpful and beneficial for personal recovery. Positive effects comprised improvement of adherence and clinical outcome as well as avoidance of decompensation and formal coercion. One-third to two-third of the patients approved informal coercion independently of their own experience (14, 19). Lucksted and Coursey showed that retrospectively some participants understood forced treatment to be in their best interest although they reported negative effects from it and wished to maintain the right to refuse treatment (37). These findings underline the controversy regarding informal coercion, which was also outlined by Norvoll and Pedersen where participants described informal coercion as part of a gray zone and only a few found it to be helpful for their mental health (27). Patients and other stakeholders with critical attitudes toward coercion would decidedly challenge the use of informal coercion at all and emphasize the importance of the reciprocal therapeutic relationship (38). The representativeness of the patients included in the studies of this review has to be considered as limited. It mostly comprises individuals who were in treatment within the mainstream public mental health-care systems (mostly institutional) rather than complementary, private, or other services. Also, the study patients consented to participate in the studies what implies a certain willingness to cooperate with the services. This is commonly considered a major limitation for representativeness of study participants in research on coercion in psychiatry.

Albeit, most professionals tended to avoid the use of informal coercion due to the ethical problems attached to interventions utilizing (formal and informal) coercion, and staff underlines the effectiveness of informal coercion to achieve better clinical outcomes in patients. In order to stay aware and reduce the use of informal coercion, continuous discussions on the issue and supervisions are rated to be helpful. However, it has to be assumed that many situations in which informal coercion is applied in routine practice the acting clinician would not be aware of using informal coercion (22). Corresponding to the included studies on patients’ perspective, professionals’ study participants might not be representative for mental health professionals in general. The willingness to participate in a study on the issue of coercion might be higher in professionals who are prone to critically reflect on delicate subjects as well as their own attitudes and routines.

### Clinical Relevance of Informal Coercion

Our review revealed that no study evaluated the clinical effects of informal coercion as a primary outcome in an appropriate study design. This may be due to methodological as well as ethical problems attached to such a study. Psychiatric treatment is a complex and multifaceted process including many factors that may help the patient to recover. It seems difficult to set up a study design that would allow for a comparison of two similar groups of patients with one undergoing a treatment process including the use of informal coercion and another receiving the same treatment without informal coercion. It might be feasible to study different therapeutic attitudes in the treatment of a selected group of patients, e.g., individuals with psychosis concerning certain decision-making processes, such as choice of medication. In this context, an operationalized negotiation process could be applied to two groups of patients with one including persuasion and inducements and one on a merely informative basis. However, this would be highly artificial and disregard the individual nature and dynamic constitution of a sound therapeutic relationship that would be the basis of a realistic decision-making process. This would be a considerable limitation for the validity of the results of such a study.

Thus, it seems comprehensible that the studies reviewed in the present article refrain to the subjective evaluation of effectiveness of informal coercion interventions by professionals and patients. Although there are several studies reporting rather positive evaluations of clinical effects in terms of fostering adherence, clinical stability, and avoiding relapse, it is not possible to draw convincing conclusions. It seems highly dependent on some process-related aspects if informal coercion is accepted by patients as beneficial for their recovery. This includes a low level of perceived coercion, high perceived fairness, and sound procedural justice (39). Mental health professionals might miss the importance of these process-related factors and tend to hold a rather utilitarian attitude toward informal coercion. Thereby, professionals are at stake to contribute to the stigma of coercion in psychiatric treatment that might lead to avoidance of the mental health system (37). The use of financial or other forms of leverage may lead to unclear responsibilities for potentially harmful medication effects, especially in the long term (25). Additionally, benefits of coercively taken medication may be extenuated by decreased satisfaction with treatment (30).

Ethical and clinical guidelines for the use of informal coercion are crucial for raising and keeping awareness on the issue similarly to formal coercion. In fact, few contemporary guidelines on the use of coercion in health care amplified their scope beyond formal coercion on informal coercion (40). Accordingly, coercive interventions including informal coercion should only be applied under the restriction of commensurability, i.e., if less invasive interventions are not available or have proven not to be effective and the expected benefit outweighs the potential harm by the intervention itself. Autonomy of the patients must always be respected and prioritized when making a decision for a treatment or care intervention. Communication and documentation has to be transparent and appropriate (40). However, applying informal coercion in an ethically, legally, and therapeutically sound procedure requires the awareness that leverage and other forms of informal coercion are very frequently used in daily mental health-care routine. Mental health professionals should, therefore, be competent to realize when they apply informal coercion and know about the impact of informal coercion as well as ethical guidelines for the use of coercion. A more prominent place for the issue of informal coercion and the therapeutic relationship in educative curricula of mental health professionals as well as more in-depth qualitative and quantitative research on informal coercion have to be strongly recommended.

### Limitations

The present systematic review provides a general overview on studies evaluating the prevalence, attitudes, and clinical effect concerning informal coercion. With respect to some important limitations, the results have to be interpreted with care. This review is merely descriptive, and no meta-analysis was intended or possible to conduct. Although the research was performed systematically, it is not known if all available publications were detected, especially the gray literature (i.e., research produced outside the academic publication channels). Most studies were conducted in the US and Europe. And although one study also included study sites in Canada, Chile, and Mexico, the results cannot be easily transferred to other countries or even regional contexts. Moreover, the methodological quality of the studies is limited, and no causal associations concerning clinical effects and consequences of informal coercion can be deducted. The use of informal coercion is supposedly interrelated with societal context, organization of the health-care system, the educational level of professionals, and many other factors that were not comprehensively controlled for in the included studies. Additionally, the representativeness of the samples was not evaluated. However, despite multiple limitations of the present review, some important aspects on informal coercion in mental health care can be concluded.

## Conclusion

This is the first review on informal coercion in mental health care. Most studies focus on leverage in general and specific leverage tools in various clinical and non-clinical contexts. Remarkably, frequent experience with informal coercion was reported by both, professionals as well as patients. The attitudes were rather positive in professionals as well as in patients at least if informal coercion was applied according to a number of procedural aspects that are also included in ethical guidelines for coercive practices in medicine (respect for patient’s autonomy, procedural fairness, and transparency in communication). There is no evidence on the clinical effects of informal coercion but subjective evaluations on potential consequences, i.e., enhancement of adherence, promotion of clinical stability, and avoidance of relapse. Negative consequences such as increasing stigma of psychiatric services, impairment of the therapeutic relationship and consequent avoidance of mental health care are considered potential adverse effects.

## Author Contributions

Conception and design; data collection, analysis, and interpretation; drafting the article and revising it critically for important intellectual content: FH and MJ.

## Conflict of Interest Statement

The authors declare that the research was conducted in the absence of any commercial or financial relationships that could be construed as a potential conflict of interest.
